# Partial volume correction for quantitative CEST imaging of acute ischemic stroke

**DOI:** 10.1002/mrm.27872

**Published:** 2019-06-14

**Authors:** Y. Msayib, G. W. J. Harston, F. Sheerin, N. P. Blockley, T. W. Okell, P. Jezzard, J. Kennedy, M. A. Chappell

**Affiliations:** ^1^ Institute of Biomedical Engineering, Department of Engineering Science University of Oxford Oxford United Kingdom; ^2^ Acute Vascular Imaging Centre, Radcliffe Department of Medicine University of Oxford Oxford United Kingdom; ^3^ Wellcome Centre for Integrative Neuroimaging, FMRIB Division, Nuffield Department of Clinical Neurosciences University of Oxford Oxford United Kingdom

**Keywords:** acute ischemic stroke, amide proton transfer, CEST MRI, partial volume correction, quantification

## Abstract

**Purpose:**

Contributions of cerebrospinal fluid (CSF) have not been previously taken into account in the quantification of APT CEST effects, and correction for the dilution of CEST effects by CSF may allow for more robust measurement of CEST signals. The objective of this study was to compare the robustness of a partial volume (PV) correction model against a standard (4‐pool) multi‐pool model as far as their ability to quantify CEST effects in healthy, normal, and pathological tissue.

**Methods:**

MRI data from 12 patients presenting with ischemic stroke, and 6 healthy subjects, were retrospectively analyzed. CEST signals derived from a 4‐pool model and a PV correction model were compared for repeatability and pathological tissue contrast. The effect of PV correction (PVC) was assessed within 3 ranges of tissue PV estimate (PVE): high PVE voxels, low PVE voxels, and the whole slice.

**Results:**

In voxels with a high tissue PVE, PV correction did not make a significant difference to absolute $APTR^{*}$. In low PVE voxels, the PVC model exhibited a significantly decreased ischemic core signal. The PVC measures exhibited higher repeatability between healthy subjects (4 pools: 3.4%, PVC: 2.4%) while maintaining a similar ischemic core CNR (0.7) to the 4‐pool model. In whole slice analysis it was found that both models exhibited similar results.

**Conclusions:**

PV correction yielded a measure of APT effects that was more repeatable than standard 4‐pool analysis while achieving a similar CNR in pathological tissue, suggesting that PV‐corrected analysis was more robust at low values of tissue PVE.

## 1 INTRODUCTION

Stroke imaging using amide proton transfer (APT) CEST has shown that tissue within the ischemic core exhibits a significant decrease in APT value.^1^, ^2^ The decrease in signal associated with pathology is similar to the reduction of APT effects that would be observed due to a dilution of CEST effects in a voxel owing to the presence of cerebrospinal fluid (CSF) that has no substantial APT effect of its own. Thus, for any application, like stroke, where a reduction in APT is indicative of pathological tissue, the contribution of CSF is a potential confound.^1^, ^3^, ^4^ In the study of [Ref. 3] it was observed that APT effects were decreased in regions around CSF, within tissue that was otherwise identified as normal‐appearing. In voxels at tissue‐CSF boundaries, such as at the peripheries of brain tissue, near the ventricles, and at fissures, possible contributions of CSF have not previously been taken into account in the analysis of CEST images.

The objective of this study was to develop a partial volume (PV) correction technique for multi‐pool model‐based analysis of APT CEST data, and to examine if there was a change in quantification of APT effects compared to the standard multi‐pool technique.

## 2 THEORY

**Figure 1:**
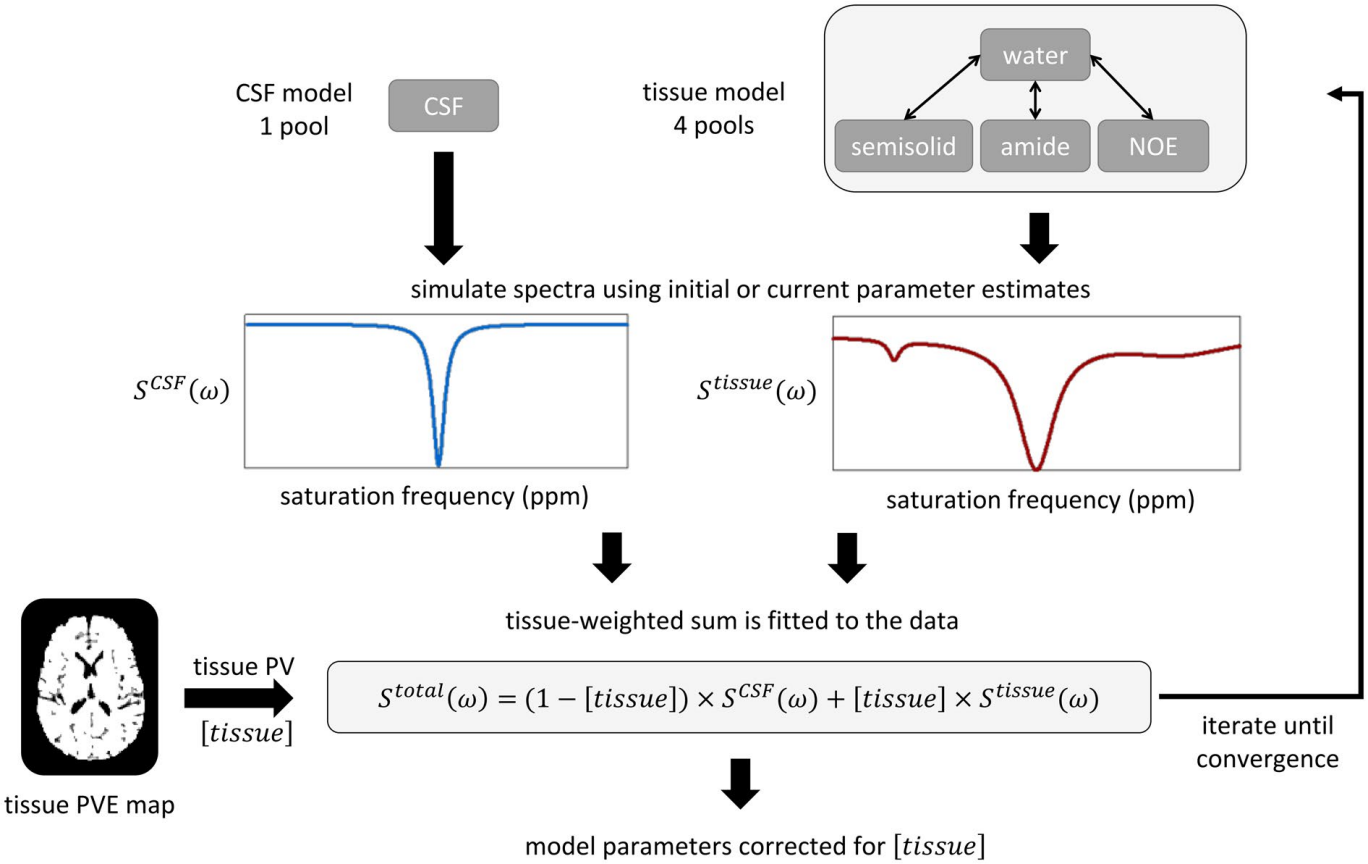
Outline of the PV correction model fitting process. The CSF and tissue model components each generate a spectrum ($S^{\mathrm{CSF}}$, $S^{\mathrm{tissue}}$), and the data are fitted to a weighted sum based on tissue PV [tissue] which is derived from a tissue PVE map

The PV correction (PVC) model is schematically illustrated in Figure 1, it is an extension of previous model‐based analysis of APT CEST data where a multi‐pool Bloch‐McConnell (BM) model of exchange was used to interpret sampled *z*‐spectra via model fitting.^5^ The PV correction model generates a tissue‐weighted sum of a *z*‐spectrum representing CSF, $S^{\mathrm{CSF}}\left(\omega \right)$, and a *z*‐spectrum representing tissue, $S^{\mathrm{tissue}}\left(\omega \right)$. The *z*‐spectra are derived from 2 separate models: $S^{\mathrm{CSF}}$ from a 1‐pool BM model of CSF, and $S^{\mathrm{tissue}}$ from a 4‐pool BM model of tissue. The weighting factor is derived from an independent estimate of tissue PV, which describes the proportion of tissue in each voxel, denoted as [tissue]. The weighted sum of the CSF and tissue components is fitted to the data:

(1) $$S^{\mathrm{total}}\left(\omega \right)=\left(1-[\text{tissue}]\right)\times S^{\mathrm{CSF}}\left(\omega \right)+[\text{tissue}]\times S^{\mathrm{tissue}}\left(\omega \right)$$

The study of [Ref. 1] found that gray matter‐white matter contrast of the APT signal, measured using the multi‐pool technique, was small. For the purpose of PV correction in this study, approximating gray matter and white matter tissue properties using a generic “tissue” description was assumed to be a suitable way to correct for CSF effects while minimizing model complexity. Model complexity is of particular consideration in this clinical imaging context, as spectral features are relatively broad and data points are limited.

## 3 METHODS

### 3.1 Study details

Six healthy volunteers (median age: 34 years) were recruited and imaged under an agreed technical development protocol approved by the institution's Research Governance Office. These volunteers underwent imaging with 4 repeated CEST scans at 3 separate time points (initial, at 24 hours and at 1 week).

Eighteen patients presenting with acute ischemic stroke were recruited into a prospective observational imaging study according to research protocols agreed by the UK National Research Ethics Service Committee (references 12/SC/0292 and 13/SC/0362) as previously described.^1^ After exclusions on the grounds of motion corruption, imaging artifacts, and secondary hemorrhage, this left 12 datasets for analysis as described in the original study. The median time from onset was 2 hours 59 minutes, 58% female, with a median patient age of 79.5 years, and a median NIHSS score at presentation of 11. Individual patient demographics are detailed in [Ref. 1].

### 3.2 Image acquisition

All patient scans were performed on a 3T Siemens Verio scanner using a 32‐channel head coil as described in [Ref. 1]. Briefly, each patient underwent a $T_{1}$‐weighted MP‐RAGE structural scan, diffusion weighted imaging (DWI) in 3 directions, multiple post‐labeling delay vessel‐encoded pseudocontinuous arterial spin labeling perfusion‐weighted imaging, and single‐slice CEST imaging with voxel dimensions $3.4\;\times \;3.4\;\times \;5.0\;{\text{mm}}^{3}$. The single‐slice CEST imaging plane was localized by a clinician based on the DWI lesion at the time of scanning. Pulsed CEST preparation was performed with 50 Gaussian pulses at a flip angle of $184^{\circ }$ (20 ms pulse, 20 ms delay) to achieve an average $B_{1}$ power of 0.55 μT. CEST preparation was applied at 32 saturation frequencies from −4.5 ppm to 4.5 ppm, and 300 ppm.^1^, ^6^ Crusher gradients were applied before readout to spoil the residual transverse magnetization. A spin‐echo echo‐planar imaging readout (*TR* = 5 s, *TE* = 23 ms, 64 × 64 matrix size, 6/8 partial Fourier) was performed after the CEST preparation pulses. The total acquisition time for the CEST sequence was 2 minutes 45 seconds. A DWI (at 24 hours) and/or $T_{2}$‐weighted FLAIR (at 1 week) follow‐up scan to enable the definition of tissue outcome was taken.

### 3.3 Processing

Image processing and analysis was performed using the FMRIB Software Library (FSL)^7^, ^8^ and MATLAB (Mathworks, Inc., Natick, Massachusetts).

#### 3.3.1 Image processing

The Brain Extraction Tool in the FSL package^9^ was used to remove the skull and non‐brain areas in all of the collected data. All of the imaging modalities were transferred to the structural space, and within time point image registration was performed using FMRIB's Linear Image Registration Tool (FLIRT). Across time point image registration was done using FMRIB's Non‐linear Image Registration Tool (FNIRT) for patients, and FLIRT for healthy subjects, both available in the FSL package.^8^, ^10^, ^11^ The different CEST frequency offsets were motion‐corrected using linear co‐registration to the unsaturated acquisition.

#### 3.3.2 Generation of partial volume estimate (PVE) maps

Tissue PV estimate (PVE), [tissue] (a fraction in the range 0.00‐1.00), was obtained using the FSL brain segmentation tool (FAST) operating on a structural $T_{1}$‐weighted scan.^12^ This generated tissue PVEs of gray matter, white matter, and CSF. For the tissue PVE map used in model fitting, gray matter and white matter PVEs were summed to give a *tissue* (*GM*+*WM*) PVE map. The images were transformed to the resolution of the CEST images by first transforming CEST data to the $T_{1}$‐weighted structural scan using the FSL tool (FLIRT),^8^, ^9^, ^10^ inverting the transformation matrix, and using that to transform the PVE maps to the resolution of the CEST images using the FSL tool applywarp.

#### 3.3.3 Region of interest (ROI) definitions in native space

In healthy subjects, the ROIs used were a whole slice mask, voxels with a high estimate of tissue PV, and voxels with a low estimate of tissue PV, defined as follows. Thresholds were applied to the tissue PVE map (defined above) to create healthy subject masks in the data space:

1. Whole slice mask: voxels with a tissue PVE between 0.50–1.00.
2. Low tissue PVE mask: voxels in the bottom half of the whole slice PVE range (0.50–0.75).
3. High tissue PVE mask: voxels in the top half of the whole slice PVE range (0.75–1.00).

In stroke patients, these PVE masks were applied to the contralateral and ischemic core ROIs. Infarct at presentation was defined using semi‐automated delineation of ADC below an externally validated threshold of $620\;\times \;10^{-6}\;{\text{mm}}^{2}/s$.^13^ Final infarct was defined preferentially on the 1 week FLAIR image, or, if not available, the *b* = 1000 DWI at 24 hours.^14^ The mask representing perfusion deficit was generated using a threshold approach where voxels with a cerebral blood flow (CBF) threshold of less than 20 ml/100 g/min were identified and clustered, and then used as a guide for manual delineation by an expert clinician (GH).^1^ Pathological ROIs used for defining the contralateral mask were: ischemic core, infarct growth (within the final infarct, but not within the presenting infarct), and oligaemia (tissue present in the perfusion deficit but not the final infarct). The tissue ROIs analyzed in this study were:

1. Ischemic core: within both presenting and final infarct definitions.
2. Mirrored contralateral mask: contralateral ROIs were obtained by non‐linearly registering the pathological masks to standard *MNI*152 space, reflection in the sagittal plane, and transforming back to CEST space.

These ROI definitions are in keeping with those used in [Ref. 1] but have been updated to improve ROI fidelity with tissue fate.^14^

#### 3.3.4 Model fitting

A continuous wave approximation of the multi‐pool Bloch‐McConnell model, including both 4 pools and PV correction, was fitted to the data^15^, ^16^, ^17^ using the implementation in the FSL tool BayCEST, which uses the variational Bayes FABBER model‐fitting routine.^3^, ^16^, ^18^ The CEST data were compensated for $B_{0}$ inhomogeneity via a variable in the model‐fitting algorithm that accounted for water resonance shift.

The quantification techniques compared in this study were 4‐pool $APTR^{*}$, and 4‐pool $APTR^{*}$ incorporating PV correction (PVC $APTR^{*}$). Defining *S* as the model‐fitted CEST spectrum of the water pool ($S_{w}$) or the water+amide pool ($S_{w\;+\;a}$) evaluated at 3.5 ppm, then, 4‐pool $APTR^{*}$:

(2) $$\left(S_{w}^{4-pool}-S_{w+a}^{4-pool}\right)/S_{0}^{4-pool}$$

which is an extension of the 3‐pool metric used in.^3^, ^16^ The 4‐pool model used in this study has been shown to exhibit higher repeatability when quantifying APT effects in clinical data, compared to more approximate 3‐pool model.^5^ The 4‐pool model comprised a pool for water, APT effects at 3.5 ppm, symmetric semisolid effects, and nuclear Overhauser effects. PVC $APTR^{*}$ was defined as follows:

(3) $$\left(S_{w}^{\mathrm{tissue}}-S_{w+a}^{\mathrm{tissue}}\right)/S_{0}^{\mathrm{tissue}}$$

For PVC $APTR^{*}$, tissue was modeled by 4 pools, and CSF was modeled by a single pool (parameter priors listed in Supporting Information Table S1). Both model fitting approaches are implemented in *Quantiphyse*, made available for download at www.quantiphyse.org.^19^

For the PV correction model, voxels with a tissue PVE of less than 50% were deemed non‐interpretable, therefore only a CSF pool was fitted for these voxels and they were not included in the further analyses. CSF pool concentration $M_{0}^{\mathrm{CSF}}$ was fixed in relation to tissue concentration using a proportionality constant, representing the mean ratio of CSF $M_{0}$ to tissue $M_{0}$ (a proton density ratio modulated by $T_{1}$). This was done as otherwise the fitting process simply reduced to the tissue model, as it alone was capable of achieving a good fit to the data. The CSF‐to‐tissue proportionality constant (0.53 ± 0.06) was estimated using the unsaturated acquisitions from the healthy subjects.

### 3.4 Analysis

The analysis plan was to define the reproducibility and contrast characteristics of healthy and pathological tissues using the 2 $APTR^{*}$ quantification techniques. All analyses were done in the native space of the data. The analyses were done for voxels with a high tissue PVE, voxels with a low tissue PVE, and the whole slice.

#### 3.4.1 Comparison of absolute $APTR^{*}$

Absolute values of $APTR^{*}$ in healthy subjects, patient contralateral tissue, and the ischemic core ROI, were compared between the 4‐pool and PVC models using one‐way analysis of variance (ANOVA).

#### 3.4.2 Repeatability

The repeatability of each technique between time points and between individuals was assessed separately in healthy subjects and in patient contralateral tissue. Repeatability was quantified using the coefficient of variation (CoV, standard deviation divided by the mean).

#### 3.4.3 Spatial variability

Spatial variability (CoV within an ROI) was used as a measure of an ROIs spatial heterogeneity and was found in healthy subjects and patient contralateral tissue.

#### 3.4.4 Contrast‐to‐noise ratio (CNR)

CNR of the ischemic core was defined as follows. Ischemic core contrast was defined as the difference in mean signal between the ischemic core ROI ($m_{\mathrm{IC}}$) and the contralateral ($m_{\mathrm{CO}}$) ROI, and noise was defined as the standard deviation (SD) in the contralateral ROI ($SD_{\mathrm{CO}}$): $\text{CNR}\;=\;\left(m_{\mathrm{IC}}-m_{\mathrm{CO}}\right)/SD_{\mathrm{CO}}$.

#### 3.4.5 Variability of $APTR^{*}$ across PVE ranges

The dependence of the 4‐pool and PVC measures on tissue PVE was assessed by finding the CoV across 10 equally spaced PVE ranges of CSF from 0% to 50%.

#### 3.4.6 Statistical analysis

Comparisons between multiple groups (ROIs and/or quantification techniques) were preceded with a one‐way ANOVA test for significance, and *post hoc* pairwise testing was done using a 2‐tailed Welch's unequal variances *t*‐test with $\alpha_{\mathrm{crit}}\;=\;0.05$, corrected for multiple comparisons using the Bonferroni method.

## 4 RESULTS

### 4.1 Subject $APTR^{*}$ maps

**Figure 2:**
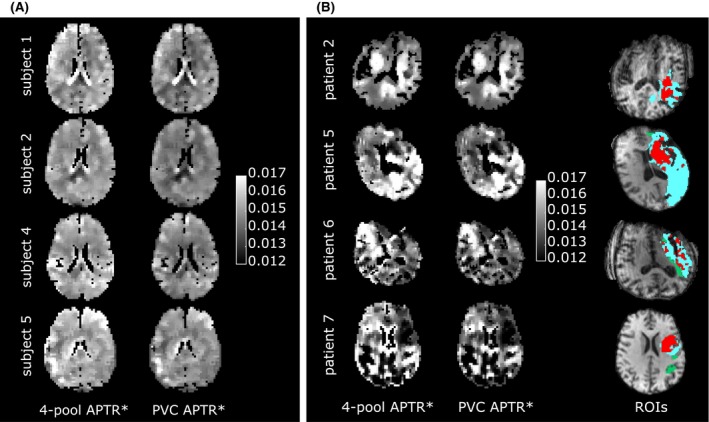
A, Healthy subject $APTR^{*}$ maps, and B, representative patient maps using 4‐pool and PVC models. Red: ischemic core, green: oligaemia, cyan: infarct growth

Healthy subject and patient $APTR^{*}$ maps are shown in Figure 2. Areas of $APTR^{*}$ hyperintensity at the periphery of the brain on the 4‐pool maps were diminished on the PVC maps, where $APTR^{*}$ was generally more uniform (see healthy subject slices in Figure 2A in particular). In stroke patients the PVC maps showed little overall change. In some cases (patients 6 and 7) the extent of hyperintense regions at tissue‐CSF boundaries was smaller when compared to 4‐pool $APTR^{*}$.

### 4.2 $APTR^{*}$ in voxels with a high tissue PVE

**Figure 3:**
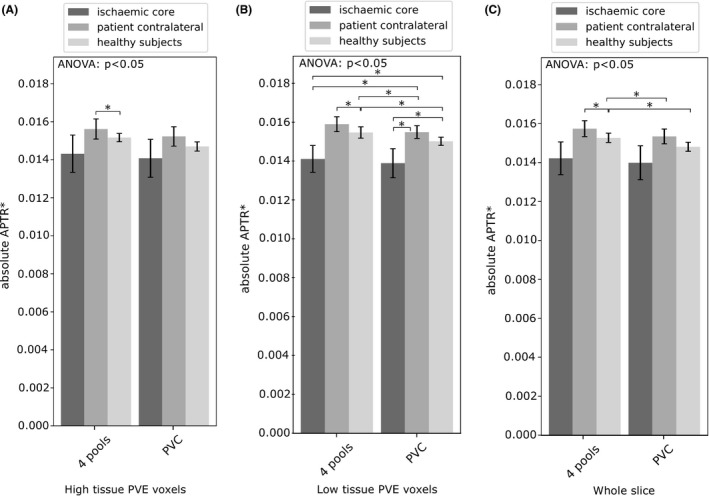
Subject mean absolute $APTR^{*}$ using the 4‐pool and PVC measures, shown for A voxels with a high tissue PVE, B voxels with a low tissue PVE, and C the whole slice. Error bars are the 95% CI. Statistical significance between ROIs is denoted by an asterisk. Significant results using the same ROI are highlighted with a solid line

**Figure 4:**
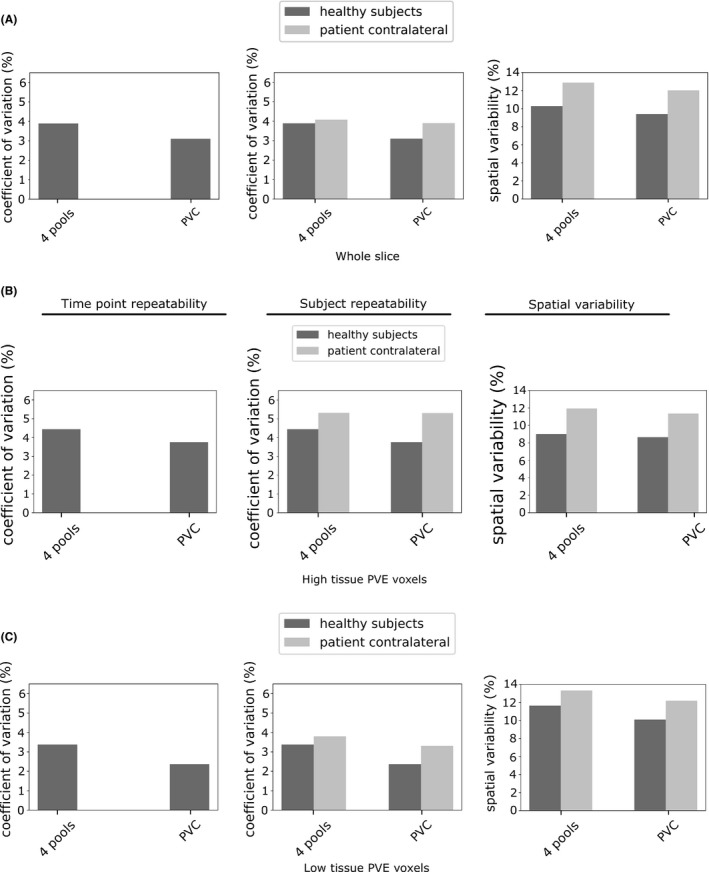
Time point repeatability (left), subject repeatability (center), and spatial variability (right), using the 4‐pool and PVC models. Shown for A, voxels with a high tissue PVE, B, voxels with a low tissue PVE, and C, the whole slice

The mean value of $APTR^{*}$ in subjects, using the 4‐pool and PV correction models, is shown in Figure 3A. The values were, as expected, almost identical for both models. Repeatability between time points and subjects is shown in Figure 4A (mean and SD reported in Table 1A). In healthy subjects, repeatability between time points was similar between the 2 models (4 pools: 2.3%, PVC: 2.2%), and repeatability between patients was identical (5.3%). Repeatability between healthy subjects was slightly higher using the PVC measure (4 pools: 4.4%, PVC: 3.8%). The PVC measure exhibited slightly lower spatial variability in healthy subjects (4 pools: 8.5%, PVC: 8.0%) and in patients (4 pools: 12.0%, PVC: 11.4% (mean and SD reported in Table 1B). Ischemic core CNR was 0.59 using the 4‐pool model, and 0.55 using PVC.

**Table 1:** A, Time point repeatability and subject repeatability in healthy subjects and patient contralateral tissue. (b) Spatial variability in healthy subjects and patient contralateral tissue. The mean CoV (%) is presented, with the SD in parentheses where applicable

		Healthy subjects		Patients	
		4‐pool $APTR^{*}$	PVC $APTR^{*}$	4‐pool $APTR^{*}$	PVC $APTR^{*}$
(a) Repeatability					
Time points	High PVE	2.3 (3.9)	2.2 (3.2)	‐	‐
	Low PVE	1.9 (2.5)	1.5 (1.7)	‐	‐
	Whole slice	2.0 (3.4)	1.7 (2.6)	‐	‐
Subjects	High PVE	4.4 (3.8)	3.8 (2.9)	5.3	5.3
	Low PVE	3.4 (2.7)	2.4 (1.6)	3.8	3.3
	Whole slice	3.9 (3.4)	3.1 (2.4)	4.1	3.9
(b) Spatial variability					
High PVE	8.5 (2.0)	8.0 (1.5)	12.0 (2.9)	11.4 (2.6)	
Low PVE	12.3 (2.3)	10.5 (2.2)	13.3 (4.8)	12.2 (4.2)	
Whole slice	10.3 (1.7)	9.2 (1.6)	12.9 (3.9)	12.0 (3.4)	

### 4.3 $APTR^{*}$ in voxels with a low tissue PVE

In voxels with a low tissue PVE, the absolute value of $APTR^{*}$ (Figure 3B) was, in healthy subjects, significantly different between the 4‐pool and PVC models (4 pools: 0.0155 ± 0.0003, PVC: 0.0150 ± 0.0002). Using the PVC model, the ischemic core ROI (0.0139 ± 0.0008) was significantly lower than the patient contralateral ROI (0.0155 ± 0.0003), whereas a significant difference was not present using the 4‐pool model (ischemic core: 0.0141 ± 0.0007, contralateral: 0.0159 ± 0.0004). Repeatability (Figure 4B) between time points (4 pools: 1.9%, PVC: 1.5%) and healthy subjects (4 pools: 3.4%, PVC: 2.4%) was higher using the PVC model, as was repeatability assessed between patients (4 pools: 3.8%, PVC: 3.3%). Spatial variability (Figure 4B) was 13.3% using the 4‐pool measure, and 12.2% using PVC. Ischemic core CNR was similar between the models: 0.69 using 4‐pool analysis, and 0.68 using the PVC model.

### 4.4 $APTR^{*}$ in the whole slice

Mean $APTR^{*}$ was calculated over the whole slice (ie, low tissue PVE voxels ⋃ high tissue PVE voxels). In healthy subjects, the non‐PV corrected measure (0.0153 ± 0.0002) was significantly different from the PVC measure (0.0148 ± 0.0002). Neither model exhibited a significant difference in mean $APTR^{*}$ between the ischemic core and patient contralateral tissue. Repeatability between time points (Figure 4C) was 2.0% using the 4‐pool measure and 1.7% using the PVC measure. Between healthy subjects, repeatability was 3.9% using the 4‐pool measure and 3.1% using the PVC measure (4.1% and 3.9% between patients). The 4‐pool measure exhibited a spatial variability of 10.3% in healthy subjects, and the PVC measure exhibited a value of 9.2%. The CNR of the ischemic core was 0.59 using 4‐pool analysis, and 0.56 using the PVC model. Four‐pool $APTR^{*}$ was relatively invariant to the CSF percentile (0.84%). After PV correction, the signal exhibited lower variance across CSF percentiles (0.75%).

## 5 DISCUSSION

In voxels with a low tissue PVE, the PVC measure exhibited higher repeatability and spatial uniformity of $APTR^{*}$ compared to the 4‐pool measure, while preserving a similar ischemic core CNR. In whole slice analysis, PV correction yielded a small and consistent decrease in spatial variability. Four‐pool $APTR^{*}$ did not vary strongly as a function of voxel CSF fraction, and this was lower still after PV correction was applied.

In voxels with a high tissue PVE, the 4‐pool and PVC models did not differ significantly in mean $APTR^{*}$ and exhibited similar repeatability measures, indicating that voxels in this range did not benefit significantly from PV correction. Voxels with a low tissue PVE are more vulnerable to a dilution of CEST effects by CSF, yet in these voxels the PVC model was able to identify a significant decrease in the ischemic core $APTR^{*}$. The effect of PV correction on voxels with a low tissue PVE was observed as an increase in time point repeatability, subject repeatability, and spatial homogeneity, compared to the 4‐pool model. This was achieved without substantially affecting CNR of pathological tissue. The PVC measure also exhibited less dependence on tissue PV fraction than that of the 4‐pool model, although in both cases the dependence was weak. The PV correction model, therefore, appears to provide more robust quantification of $APTR^{*}$ in voxels that have a low tissue PVE compared to a non‐PV‐corrected analysis. Compared to normal tissue, pathological tissue might not exhibit the same voxel intensity characteristics that distinguish the different tissue classes, and it can therefore be more difficult to derive PVE maps from stroke patient data than in healthy subjects. This leads to potential inaccuracy of the segmentation method in the affected regions, particularly in the low PVE range. The robustness measures evaluated in healthy subject data were not subject to this potential source of inaccuracy. Whole slice analysis indicated that, on balance, the 2 models exhibited similar measures of repeatability. This was reflected in the visual comparison of subject slices where, although the $APTR^{*}$ maps from healthy subjects displayed some improvement, images from patients showed little overall change. In the latter case residual partial volume effect appears to have remained after PV correction.

Significant differences in $APTR^{*}$ between the patient contralateral and healthy subjects were observed exclusively in the non‐PV corrected measure. The geriatric patient population imaged in this study exhibits a degree of cortical tissue atrophy compared to the healthy volunteers, making the patient results more susceptible to partial volume effects. The non‐PV corrected measure does not account for the partial volume distribution of CSF, whereas the PV corrected measure does, which may be why the former exhibits statistically significant differences between patient and healthy subject ROIs, whereas the latter does not. This explanation is especially pertinent to the low tissue PVE voxels. As to why the high tissue PVE voxels also exhibit a significant difference, it should be noted that while the high tissue PVE voxels contain little CSF contribution by definition, $APTR^{*}$ in these voxels is nevertheless influenced by surrounding voxels which may have a low PVE via the spatial regularization that occurs in the analysis.

## 6 CONCLUSION

PV correction yielded a measure of APT effects that was more repeatable than standard 4‐pool analysis while achieving a similar CNR in pathological tissue, suggesting that PV‐corrected analysis was more robust at low values of voxel tissue fraction. When the whole slice was considered, application of PV correction did not make a large difference. This method of PV correction could be used for more robust quantification of CEST effects in the presence of large CSF contributions, although it might not be required in a clinical context where a simpler model, which does not correct for tissue PV effects, yields comparable results.

## DATA STATEMENT

The underlying data associated with figures in this study are available from the Oxford University Research Archive (ORA‐Data) (DOI: 10.5287/bodleian:9RmBjG1aV). For enquiries regarding the clinical data, contact JK (james.kennedy@rdm.ox.ac.uk).

## Supporting information


**TABLE S1** CSF pool priors expressed as a mean and SD. Glossary–$M_{0}$: pool concentration relative to water pool (water pool $M_{0}$ is absolute), $k_{\mathrm{ex}}$: pool→bulk water exchange rate, $T_{1}$: longitudinal relaxation time, $T_{2}$: transverse relaxation time, Δω: chemical shift with respect to water pool. ${\;}^{†}$Based on [Ref. 20], ${\;}^{‡}$Based on [Ref. 21]
