# Supplementary material for: Partial volume correction for quantitative CEST imaging of acute ischemic stroke
Source: Magn Reson Med. 2019 Jun 14;82(5):1920–8. doi: 10.1002/mrm.27872 (PMC6771886; doi:10.1002/mrm.27872)
Supplement: Supplementary file 1 — TABLE S1 CSF pool priors expressed as a mean and SD. Glossary–M0: pool concentration relative to water pool (water pool M0 is absolute), kex: pool→bulk water exchange rate, T1: longitudinal relaxation time, T2: transverse relaxation time, Δω: chemical shift with respect to water pool. †Based on [Ref. 20], ‡Based on [Ref. 21] [file MRM-82-1920-s001.pdf]

Supporting Information

Supporting Information Table S1: CSF pool priors expressed as a mean and SD. Glossary – M_0: pool concentration relative to water pool (water pool M_0 is absolute), k_{ex}: pool→bulk water exchange rate, T_1: longitudinal relaxation time, T_2: transverse relaxation time, $\Delta\omega$: chemical shift with respect to water pool. † Based on ref. (1), ‡ Based on ref. (2)

	CSF pool of the PV correction model	
	water	
Parameter	mean	SD
M_0 (norm.)	$0.527 \times M_0^{tissue}$	1×10^{-6}
k_{ex} (Hz)	—	—
T_1 (s)	1.9†	0.15
T_2 (ms)	250‡	50
$\Delta\omega$ (ppm)	0	1×10^{-6}

References

[1] Hashemi R. H., Bradley W. G., Listanti C. J.. *Magnetic Resonance Imaging: The Basics*. Philadelphia, USA: Lippincott Williams & Wilkins3 ed. 2010.

[2] Carneiro A. A. O., Vilela G. R., Araujo D. B. de, Baffa O.. MRI Relaxometry: Methods and Applications *Brazilian Journal of Physics.* 2006;36:9–15.
